# Examining a Group Acceptance and Commitment Therapy Intervention for Music Performance Anxiety in Student Vocalists

**DOI:** 10.3389/fpsyg.2020.01127

**Published:** 2020-05-29

**Authors:** Laura K. Clarke, Margaret S. Osborne, John A. Baranoff

**Affiliations:** ^1^Melbourne School of Psychological Sciences, The University of Melbourne, Melbourne, VIC, Australia; ^2^Melbourne Conservatorium of Music, The University of Melbourne, Melbourne, VIC, Australia; ^3^Centre for Treatment of Anxiety and Depression, SA Health, Adelaide, SA, Australia; ^4^University of Adelaide, Adelaide, SA, Australia

**Keywords:** performance anxiety, psychological flexibility, wellbeing, musicians, early intervention, ACT, conservatorium, performance psychology

## Abstract

Music performance anxiety (MPA) is a distressing and persistent anxious apprehension related to musical performance. The experience of MPA forces many musicians to give up performing or develop maladaptive coping mechanisms (e.g., avoidance or substance use), which can impact their career and wellbeing. High levels of MPA in students and vocalists are reported in the literature. Vocalists present a unique challenge for clinicians in that vocal and breathing mechanisms, required for performance, are negatively impacted when anxious. Acceptance and commitment therapy (ACT) has demonstrated efficacy for the treatment of a range of psychological problems including social anxiety disorder (of which MPA may be indicated as a subtype). This study sought to investigate whether group-based ACT may be a feasible and effective intervention for MPA in Australian student vocalists and aimed to design an intervention that could be adopted by music education providers. Potential participants (*N* = 31) completed an online survey including demographic questions and outcome measures. Six vocal students (four females; two males; aged *M* = 20.33 years) with elevated MPA scores participated in the ACT for MPA group program and 3-month follow-up. Group sessions were 2 h each week for six consecutive weeks. Participants were followed up 3 months post-intervention via online survey. There was a significant increase in psychological flexibility and significant decreases in MPA and psychological inflexibility. Gains were maintained at 3-month follow-up. The current study offers preliminary evidence for the feasibility and effectiveness of a group-based ACT protocol for musicians with performance anxiety which may be incorporated into tertiary performance training curricula.

## Introduction

Music performance can be thrilling and satisfying; however, for many performers who experience music performance anxiety (MPA) the stage can be frightening and threatening, leading many to give up performing or develop maladaptive coping strategies (e.g., substance use), which can impact their career and wellbeing (Kenny, 2011; Braden et al., 2015).

### Music Performance Anxiety

Music performance anxiety is a distressing and persistent anxious apprehension related to musical performance (Kenny, 2011). Symptoms of MPA may be cognitive (e.g., worry, narrowed attention focusing on mistakes and/or physiological symptoms, and perceived threat) or physiological (e.g., shallow breath, heart palpitations, dry mouth, tension, and shaking; Stothert, 2012; Juncos et al., 2017). The experience of MPA may also involve behavioral avoidance (e.g., avoiding auditions or solos), subjective stress, and occupational impairment (Stothert, 2012; Juncos et al., 2017). Performance anxiety is listed as a specifier for the diagnosis of social anxiety disorder (SAD) in the diagnostic and statistical manual for mental disorders (DSM-5; American Psychiatric Association, 2013), and it has been proposed that MPA may be a specific subtype of performance anxiety (Kenny, 2011). MPA is usually more severe when there is a perceived evaluative threat (an audience), high ego investment, or fear of failure, but it can occur in any performance setting (Kenny, 2011). It is prevalent across the lifespan and affects both professional and student musicians alike (Kenny et al., 2014a; Braden et al., 2015; Juncos and Markman, 2016; Juncos et al., 2017).

High levels of MPA are reported in the literature investigating MPA in students, many of whom report that MPA negatively affects their ability to perform and that they need help with MPA-related problems (Tamborrino, 2001; Kaspersen and Gotestam, 2002; Williamon and Thompson, 2006). A large portion of faculty (65%) and students (80%) at a school of music within a major Midwestern University in the United States believed that performance anxiety prevention and reduction should be addressed in music curriculum (Tamborrino, 2001). Choral musicians also suffer high levels of MPA (Ryan and Andrews, 2009; Stothert, 2012), yet vocalists, and in particular student vocalists, are under-represented in the MPA literature. Vocalists with MPA present a unique challenge for clinicians in that vocal performance relies on vocal and breathing mechanisms, both of which are negatively affected when the performer is anxious (Juncos et al., 2017). A survey of community choral singers found that 95% experienced MPA symptoms, most commonly hyperventilation, tension, and dry mouth (Stothert, 2012). Such symptoms may adversely impact vocal performance through compromised breath support, reduced amount of air affecting vocal tone quality, and hyperventilation causing light-headedness which may pose a risk on stage. Additionally, tension prevents the vocal mechanism from functioning properly and dry mouth can affect diction (Stothert, 2012).

### Current Interventions for Music Performance Anxiety

There is limited evidence of methodologically rigorous intervention for MPA in the literature (Kenny, 2005), which mostly includes pharmacological and psychological treatments. A significant number of musicians report using medication, such as beta blockers, to manage their MPA (Kenny et al., 2014a). Taken in low doses, beta blockers effectively lower physiological symptoms of MPA (Gates et al., 1985); however, pharmacotherapy is typically contraindicated in the treatment of performance anxiety because it interferes with exposure therapies and the extinction of fear responses (Birk, 2004). Researchers have cautioned against the use of beta blockers in singers due to the impact on respiratory exertion (Patston and Loughlan, 2014). High doses of beta blockers may also impair performance ability (Gates et al., 1985). Furthermore, beta blockers are likely to be of limited benefit to musicians with MPA symptoms that are largely cognitive or emotional, as beta blockers target only physiological symptoms (Kenny, 2011).

Psychotherapy presents a viable and effective treatment option for debilitating MPA, without the potential for negative physiological side-effects compromising musical technique. Psychodynamically oriented therapy, targeting unresolved complex emotions and defenses arising from ruptures to early attachment relationships, has shown promise for the treatment of severe forms of MPA (Kenny et al., 2014b, 2016; Kenny and Holmes, 2015; Kenny, 2016b). Cognitive behavior therapy (CBT) is commonly utilized in MPA treatment research (Clark and Agras, 1991; Osborne et al., 2007; Braden et al., 2015; Kenny and Halls, 2018). Across systematic reviews (see Kenny, 2005; Burin and Osorio, 2016), there is evidence for the effectiveness of CBT interventions for MPA, with a main effect being reduction of MPA symptoms (demonstrated in 10 out of 19 studies). However, positive findings are not unanimous, and some studies do not show favorable results (Burin and Osorio, 2016). Additionally, there are methodological weaknesses across studies, including small sample sizes, lack of standardized techniques and duration of treatment, non-statistical analyses of results, and lack of information about MPA levels experienced by participants (Kenny, 2005; Burin and Osorio, 2016).

Cognitive behavior therapy focuses heavily on symptom alleviation, working within a mastery and control framework where the main goal is to teach clients how to gain control over their anxiety and related symptoms (Eifert and Forsyth, 2005). Unfortunately, attempts to control or suppress thoughts and feelings can paradoxically increase the experience of those thoughts and feelings (Eifert and Forsyth, 2005; Brooks, 2014). Additionally, it has been proposed that it is not simply the experience of physiological symptoms of anxiety, but an individual’s *appraisal* of these symptoms, which impacts performance (Brooks, 2014). Indeed, individuals who reappraise anxiety as excitement demonstrate improved performance (Brooks, 2014). Attempts to control or suppress thoughts and feelings may reinforce the notion that physiological symptoms are something negative, to be eliminated (Brooks, 2014).

### Acceptance and Commitment Therapy

Acceptance and commitment therapy (ACT) is a multicomponent approach incorporating acceptance, mindful awareness of experience, values clarification, and various strategies from traditional behavior therapy such as exposure (Levin et al., 2017). Theoretically, psychological suffering derives from *psychological inflexibility*, a core pathological process wherein behavior is rigidly guided by immediate psychological experiences rather than by the individual’s values or goals (Levin et al., 2017). A major component of psychological inflexibility is *experiential avoidance*, wherein individuals avoid negative internal experiences such as thoughts, emotions, bodily sensations, and memories (Eifert and Forsyth, 2005; Kocovski et al., 2013). It is proposed that in SAD (of which MPA can be considered a circumscribed subtype), individuals engage in avoidance of psychological and emotional experiences that occur in the context of social situations (Eifert and Forsyth, 2005) and these avoidance behaviors interfere with values-guided action (Kocovski et al., 2013).

The central premise of acceptance-based psychological interventions is that “the struggle to be without distress is the problem, not the presence of these thoughts and feelings” (Gardner and Moore, 2007, p.73). ACT aims to increase *psychological flexibility* (“the ability to be in the present moment with full awareness and openness to our experience, and to take action guided by our values”; Harris, 2009, p. 12), which is made up of six interrelated components, collectively referred to as the ACT hexaflex (Hayes et al., 2006). These components are: (i) contact with the present moment; (ii) defusion; (iii) acceptance; (iv) self as context; (v) values; and (vi) committed action (Hayes et al., 2006; Harris, 2009). Strategies related to these components are used to promote acceptance of unwanted internal experiences and increase values-guided action (Kocovski et al., 2013).

Two randomized controlled trials investigated the use of ACT as a treatment for SAD, finding that ACT outperformed control and was as efficacious as CBT (Kocovski et al., 2013; Craske et al., 2014). The evidence base for ACT more widely continues to grow, as reflected in the current edition of Evidence-Based Psychological Interventions in the Treatment of Mental Disorders, published by the Australian Psychological Society (Australian Psychological Society, 2018). ACT demonstrates level II evidence (“a study of test accuracy with an independent, blinded comparison with a valid reference standard, among consecutive persons with a defined clinical presentation”; p. 11) for many disorders, in particular for anxiety disorders (Australian Psychological Society, 2018).

#### Acceptance and Commitment Therapy for Music Performance Anxiety

The demonstrated efficacy of ACT with a range of disorders, and in particular with SAD, suggests it may be a useful intervention for MPA. Two single-case experiments and one pilot study have investigated this, with participants across these studies demonstrating clinically significant improvement in at least one hexaflex process, improved acceptance of MPA related thoughts and sensations, and reduced MPA (Juncos and Markman, 2016; Juncos et al., 2017; Juncos and de Paiva e Pona, 2018). The researchers noted that ACT shows promise as an effective treatment option for MPA and should be studied further (Juncos and Markman, 2016). These findings suggest that ACT may be of benefit to musicians with MPA; however, all studies were based on US populations and utilized an individual treatment design (Juncos and de Paiva e Pona, 2018).

### Group-Based Interventions

Due to limited resources in applied settings, often group-based interventions are more viable than longer, individualized treatment (Glaser et al., 2009). There are also additional benefits of group-based interventions, including facilitation of individual change via group processes such as social support, group norms, identification with the group, development of social identities, and the combination of challenge and feedback in a trusting, supportive environment (Borek and Abraham, 2018). Many published ACT studies utilize a group-treatment design; one such study examined the relative efficacy of group-based ACT compared with group-based CBT for generalized anxiety disorder (GAD; Avdagic et al., 2014). Interventions were delivered in a group format across 6 weeks, involving one 2-h session per week. Individuals in both treatment conditions (CBT and ACT) showed significant improvement in reported levels of worry, depression, stress, anxiety, and quality of life from pre- to post-assessment (Avdagic et al., 2014). Gains were maintained at 3-month follow-up and both groups, at follow-up, demonstrated reliable change rates of 60% (Avdagic et al., 2014). Preliminary data from a study undertaken at the University of New South Wales showed group-based ACT was as efficacious as group-based CBT for university students with mixed anxiety problems, with ACT participants experiencing fewer anxiety symptoms than CBT participants at 12-month follow-up (see Glaser et al., 2009).

### The Present Study

Given the high frequency of debilitating MPA reported by tertiary student musicians (Tamborrino, 2001; Kaspersen and Gotestam, 2002; Williamon and Thompson, 2006) and initial empirical evidence for the effectiveness of ACT in tertiary student populations with mixed anxiety and specifically with MPA and performance issues (Glaser et al., 2009; Juncos et al., 2017), we intended to design an intervention that could be adopted by music education providers and included in curricula to assist young musicians to understand and manage MPA early in their careers. If successful, this model could potentially be adapted and implemented with a diverse population of musicians at different ages and stages of their career. Primarily, we sought to investigate whether group-based ACT may be a feasible and effective intervention for MPA in Australian student vocalists. We hypothesized that participants would demonstrate increased psychological flexibility, decreased psychological inflexibility, and decreased symptoms of MPA at the conclusion of the program. To assess the specificity of the program for MPA, secondary investigations were also conducted to determine potential transfer of therapeutic effects to depression, generalized anxiety, stress, and wellbeing.

## Materials and Methods

### Participants

Participants were a convenience sample of student vocalists enrolled in tertiary-level music studies. Thirty-one individuals (78.1% female) aged 19 to 61 years (*M* = 30.28, *SD* = 11.80), responded to an online survey (Phase 1; see section “Procedure”) with MPA scores ranging from 40 to 197 (*M* = 140.97, *SD* = 34.67). Following Phase 2 assessment (see section “Procedure”), a final group of six individuals (four females; two males; aged *M* = 20.33 years, *SD* = 1.37) participated in the full group program and follow-up.^1^ All were undergraduate students in their first (*n* = 1), second (*n* = 2), or third (*n* = 3) year of studies. Half (*n* = 3) were currently performing as amateur musicians. Total years of vocal training ranged from 8 to 14 years (*M* = 11.50, *SD* = 2.26). Participants’ prior treatment for MPA included no treatment (*n* = 2), psychological treatment (*n* = 2), physiological therapies (*n* = 2), medication (*n* = 1), and performance coaching (*n* = 1).

### Measures

#### Kenny Music Performance Anxiety Inventory (Kenny, 2009)

This 40-item self-report measure asks participants to rate on a Likert scale (0–6) the extent to which they experience various physiological, cognitive, behavioral, and emotional symptoms of MPA. Participants receive an overall score between 0 and 240, with higher scores indicating greater MPA. The Kenny Music Performance Anxiety Inventory (K-MPAI) is widely used in MPA research, including recent investigations into ACT for MPA (e.g., Juncos and Markman, 2016; Juncos et al., 2017). The scale has demonstrated excellent internal reliability (Cronbach’s alpha = 0.94; Chang-Arana et al., 2017), and convergent validity with well-established measures of trait and social anxiety (Kenny et al., 2014a). Kenny (2016a) suggests that the cut point on the K-MPAI may depend on individual issues of clinical interest. This study used the generally utilized clinical cut-off score of 105 (Ackermann et al., 2014; Juncos et al., 2017; Kenny and Halls, 2018).

#### Multidimensional Psychological Flexibility Inventory-Short Form (Rolffs et al., 2018)

The Multidimensional Psychological Flexibility Inventory (MPFI) is a 60-item measure created in response to a need for a single, comprehensive and multidimensional assessment of the 12 key components of psychological flexibility and inflexibility, as posited by ACT theory (Rolffs et al., 2018). Previous tools (e.g., AAQ; Hayes et al., 2004; AAQ-II; Bond et al., 2011; AFQ; Greco et al., 2008; Fergus et al., 2012) allowed for assessment of global levels of psychological inflexibility but failed to allow for examination of specific components (Rolffs et al., 2018). Correlational results suggest that although the psychological flexibility and inflexibility processes are related, they are separate and distinct constructs (Rolffs et al., 2018). The MPFI assesses six factors feeding into each of two meta-factors (psychological flexibility and psychological inflexibility), for example, committed action (e.g., *“Even when I stumbled in my efforts, I didn’t quit working toward what is important”*), defusion (e.g., *“I was able to let negative feelings come and go without getting caught up in them”*), avoidance (e.g., *“I tried to distract myself when I felt unpleasant emotions”*), and fusion (e.g., *“Negative thoughts and feelings tended to stick with me for a long time”*). The 24-item short form asks participants to indicate on a six-point Likert scale how much each item applied to them in the past 2 weeks. Participants receive a score from 1 to 6 for each meta-factor and each individual component.

To maintain consistency with other measures used and to make the scale performance-specific, participants in the current study were asked to rate their experiences over the previous week (rather than month), or during their most recent performance if they had not performed in the last week. Slight adaptations were made to six items on the scale to make it performance-specific; for example, where question 10 on the original MPFI-Short Form (SF), measuring values, states *“I stuck to my deeper priorities in life,”* this was modified to read *“I stuck to my deeper priorities as a performer.”* The MFPI demonstrates convergent validity via strong correlations with the most widely used measures of psychological inflexibility (i.e., the AAQ, the AAQ-II, and the AFQ-Y), and discriminant validity via low to moderate correlations with conceptually distinct constructs (e.g., emotional intelligence, neuroticism, and curiosity; Rolffs et al., 2018).

#### Depression, Anxiety, and Stress Scale (Lovibond and Lovibond, 1995)

This 21-item self-report scale asks participants to rate their experiences of various symptoms of anxiety, depression, and stress on a Likert scale (0–3), over the past week (Lovibond and Lovibond, 1995). Items for each subscale are summed, providing a total score, from 0 to 21, for each of depression, anxiety, and stress. Converted, full-measure scores are reported in results. Reliability and validity of the Depression, Anxiety, and Stress Scale (DASS-21) has been well demonstrated (Henry and Crawford, 2005; Lovibond and Lovibond, 1995; Ng et al., 2007).

#### Mental Health Continuum-Short Form (Keyes, 2005)

The Mental Health Continuum-Short Form (MHC-SF) is a 14-item self-report scale that asks participants to indicate on a Likert scale (0–5) how often they have experienced aspects of wellbeing including emotional wellbeing (e.g., “happy”), social wellbeing (e.g., “that people are basically good”), and psychological wellbeing (e.g., “that you liked most parts of your personality”). The reference period was changed from “the past month” to “the past week” in order to maintain consistency across measures. Participants receive an overall wellbeing score from 0 to 5, where higher scores indicate greater wellbeing (Keyes, 2005; Keyes et al., 2008). The MHC-SF has demonstrated moderate test-retest reliability and good convergent and discriminant validity with existing measures (Lamers et al., 2011).

#### Program Evaluation and Feedback

Post-intervention, participants completed a brief evaluation, which was developed in accordance with guidelines for collecting program evaluation data published by the University of Wisconsin Extension (Taylor-Powell and Renner, 2009). Six questions addressed satisfaction with program content, activities, and facilitators on a six-point Likert scale. The evaluation also contained five open-ended questions exploring what participants liked most and least about the program, what they feel they gained from participating, and suggestions to improve future groups. Similar open-ended questions were included in the 3-month follow-up, to allow participants to comment on their experiences of MPA and practice of skills learned since the group program ended.

### Procedure

Ethics approval was granted by The University of Melbourne. Several tertiary music education providers in Melbourne, Australia were approached to ask if they could advertise the study. Those who agreed sent an email to vocal students enrolled at their institution, containing details of the study, a link to an online survey, and a copy of the plain language statement (PLS). Advertising posters and flyers were also distributed at the Melbourne Conservatorium of Music.

#### Phase 1

Individuals who were interested in participating completed an online survey that was sent to them via email by their tertiary music education provider. The survey was created using Survey Monkey and contained a copy of the PLS, the K-MPAI, MPFI, and DASS, and questions pertaining to demographic information, performance demands, and current or previous treatment for MPA. Consent to participate was obtained via tick box at the beginning of the survey. Participants also provided consent to participate in follow-up portions of the study via check boxes and by providing their contact details. Approximately 2 weeks was given to complete the survey.

#### Phase 2

Respondents who met clinical cutoff on the K-MPAI (total score above 105) were contacted with further information about the proposed groups and invited to participate in a clinical interview. Fifteen individuals responded and were screened with the Structured Clinical Interview for DSM-5 Disorders (SCID-5-RV), modules A (mood disorders) and B (psychotic and associated symptoms). All interviews were completed via telephone at a prearranged time. Six participants were subsequently excluded from the study: five met criteria for a current mood disorder; one stated they were not a vocalist. Nine participants were invited to participate in the group program and seven confirmed their place; however, one participant withdrew after the second group session due to physical health concerns unrelated to MPA. Groups were run once per week for 2 h, for six consecutive weeks. In any instance where a participant was not able to attend a group session, an individual make-up session was organized.

##### “Acceptance and commitment therapy for music performance anxiety” group program

Sessions were led by a clinical psychologist in training who is ACT-trained and co-facilitated by a registered psychologist with over 18 years clinical experience. Sessions were video recorded and reviewed by an experienced clinical psychologist with expertise in ACT to oversee treatment fidelity. Each session concluded with instructions to participants about between-session tasks to be completed, which were reviewed at the beginning of each subsequent session. The format of each session was developed on the basis of the six-session group program adaptation for university students with mixed anxiety problems by Glaser et al. (2009), of Eifert and Forsyth’s (2005) treatment manual *ACT for Anxiety Disorders*. The manual describes concepts, principles, and techniques relevant to ACT, and provides session guidelines. Early sessions incorporate psychoeducation and orientation to treatment including exploration of willingness to engage with private experiences. All sessions include activities and exercises that aim to build ACT skills (e.g., mindfulness, acceptance, and values identification). Later sessions involve skills consolidation and skills practice via interoceptive and imaginal exposure exercises.

The ACT method encourages modification of program elements to meet specific needs (Eifert and Forsyth, 2005). In the current study, for example, interoceptive exposure activities were chosen to be appropriate for vocalists, and participants were asked specifically about their music performance values as well as their broader life values. As the current program design was group-based, it was possible to incorporate *in vivo* exposure (each group member performed a short piece, a’capella, in session five). To increase participant willingness and motivation; the exploration of values was introduced in session one, earlier than proposed by Glaser et al. (2009; session three) and Eifert and Forsyth (2005; also session three). This decision was made on the basis of research by Falk et al. (2015), who found that participants who engaged in pre-intervention self-affirmations (the process of thinking or writing about one’s core values), demonstrated increased health-related behavior change. Additionally, Glaser et al. (2009) stated in the discussion of their study that the introduction of values early in their treatment may have enhanced participants’ motivation.

At the beginning of the first group session, participants completed the MHC, DASS, and MPFI. This enabled baseline measures of psychological flexibility and inflexibility to be established. In order to maximize treatment time, the K-MPAI baseline value was established in Phase 1. Post-treatment (conclusion of session six), participants completed all outcome measures and a short program evaluation (see section “Measures”). Participants were also followed up three months post-intervention, via email containing a link to an online survey which included all outcome measures and questions about their experience in the program (see section “Measures”). A 1-week period was allowed for responses. Treatment was provided free of charge; participants were offered a $50 gift card if they participated in all aspects of the study, including 3-month follow up.

## Results

### Changes in Group Mean Scores

Repeated measures analyses of variance (ANOVA) were used to compare pre-treatment, post-treatment, and follow-up scores on the K-MPAI, MPFI, MHC, and DASS. Means, standard deviations, and inferential statistics are presented in Table 1. Given the small sample size (*N* = 6) all results should be interpreted cautiously and considered exploratory (using the G^∗^Power3.1 power analysis program, an a-priori power calculation at 0.8, medium effect size *f* = 0.25 recommends a sample size of 28; Faul et al., 2007).

**TABLE 1:** Descriptive results and group comparisons over time.

Variable	Pre-treatment *M (SD)*	Post-treatment *M (SD)*	Follow up *M (SD)*	*F*	*Partial*η ^2^
Music performance anxiety (K-MPAI)^∧+^	140.33 (18.58)	119.17 (19.42)	116.17 (21.22)	9.48*	0.66
Psychological flexibility (MPFI)^+^	3.76 (0.48)	4.36 (0.44)	4.67 (0.61)	6.74*	0.57
Psychological inflexibility (MPFI)	3.39 (0.35)	2.51 (0.48)	2.83 (0.66)	5.40*	0.52
Wellbeing (MHC)^∧#^	3.24 (0.54)	3.69 (0.35)	3.14 (0.47)	10.63**	0.68
Depression (DASS)	14.00 (9.12)	8.33 (4.63)	9.67 (6.25)	2.34	0.32
Anxiety (DASS)	13.00 (9.70)	10.00 (7.48)	8.67 (6.15)	0.51	0.09
Stress (DASS)	19.33 (5.89)	14.33 (8.80)	11.33 (3.93)	1.97	0.28

N = 6. *p = < 0.05, **p = < 0.01. *p*-value < 0.05 Bonferroni correction used for post-hoc pairwise comparisons: ∧statistically significant change from pre- to post-treatment. ^#^Statistically significant change from post-treatment to follow-up. ^+^Statistically significant change from pre-treatment to follow-up. DASS-42 full-scale equivalent scores reported.

**FIGURE 1:**
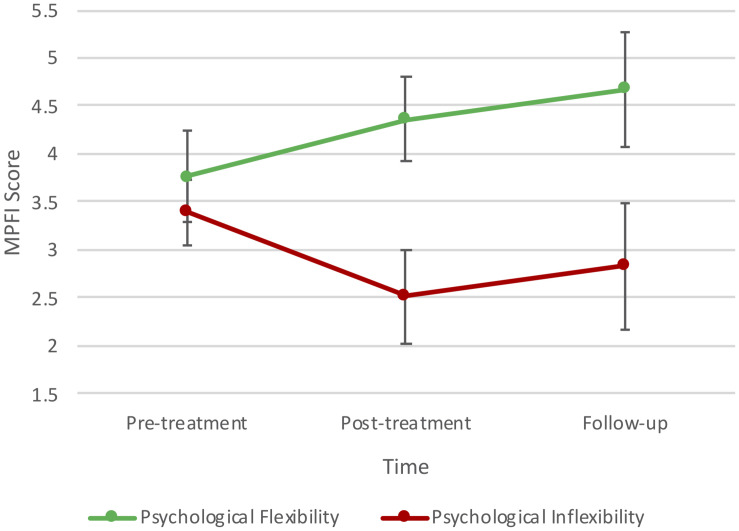
Changes in psychological flexibility and inflexibility.

As can be seen in Table 1, there were statistically significant changes in mean scores on the K-MPAI, MPFI, and MHC. A repeated measures ANOVA indicated a statistically significant decrease in mean MPA scores across time, Huynh–Feldt correction *F*(1.11, 5.58) = 9.48, *p* = 0.019, partial η^2^ = 0.655, in particular from pre- to post-treatment. Psychological flexibility scores increased across time, *F*(2, 10) = 6.74, *p* = 0.014, partial η^2^ = 0.574, in particular from pre-treatment to follow-up. There was also a statistically significant decrease in mean psychological inflexibility scores across time, *F*(2, 10) = 5.40, *p* = 0.026, partial η^2^ = 0.519. Changes in psychological flexibility and inflexibility are represented in Figure 1. Overall wellbeing scores showed a statistically significant difference between time points, *F*(2, 10) = 10.63, *p* = 0.003, partial η^2^ = 0.68, increasing from pre- to post-treatment and decreasing from post-treatment to follow-up. No statistically significant changes were observed for depression, stress, and generalized anxiety; however, reductions were clinically relevant with severity levels changing from moderate to normal levels (Lovibond and Lovibond, 1995).

### *Post hoc* Analyses

#### Changes on Dimensions of Psychological Flexibility and Inflexibility

**FIGURE 2:**
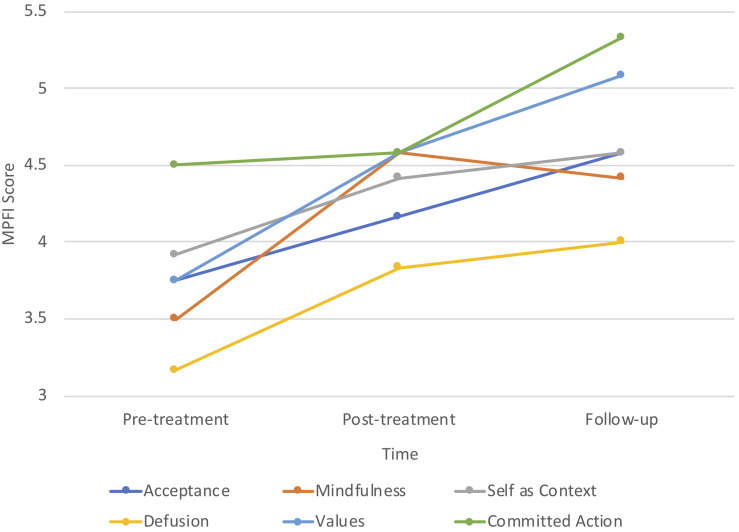
Changes on dimensions of psychological flexibility.

**FIGURE 3:**
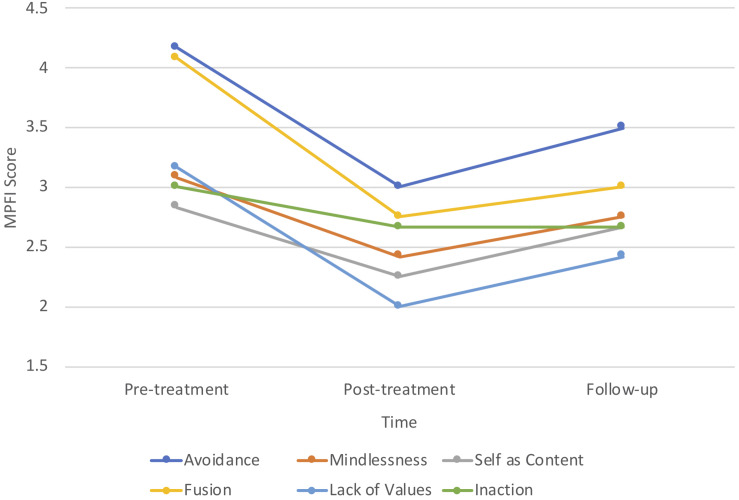
Changes on dimensions of psychological inflexibility.

While the small sample size (*N* = 6) prohibited in-depth analyses of process changes on the subscales of psychological flexibility and inflexibility, graphing the mean scores across the intervention indicated that participants demonstrated greatest flexibility in committed action and least flexibility in defusion (see Figure 2). Inversely, fusion was one of the highest subscale scores for psychological inflexibility, as was avoidance (see Figure 3).

#### Individual Change

Given the small sample size for group analysis, change in individual participants’ data on the primary outcome variables was also assessed. Jacobson and Truax (1991) offer a statistical approach to determining clinical significance, based on the assumption that clinically significant change is related to a return to normal functioning. Where normative data are unavailable, this process can be operationalized as a post-treatment score that is at least two standard deviations from the mean for that population, in the direction of functionality (Jacobson and Truax, 1991). Additionally, for the MPFI, Rolffs et al. (2018) provide statistical values for minimal detectible change (MDC; the number of points an individual must change on a scale for that change to be statistically significant). Clinical change indices for individual participants are presented in Table 2.

**TABLE 2:** Clinical change indices.

	MPA		Psychological flexibility		Psychological inflexibility	
P	Pre/Post	Pre/FU	Pre/Post	Pre/FU	Pre/Post	Pre/FU
1	−15.00^∧^	−18.00^∧^	0.58^∧^	−0.17	−0.50^∧^	−0.33^∧^
2	−10.00^∧^	−2.00^∧^	0.00	0.92^∧^	−0.17^∧^	−1.17*^+^
3	−24.00^∧^	−11.00^∧^	0.33^∧^	1.00*	−0.67^∧^	−0.08^∧^
4	−31.00^∧^	−55.00*	1.75*^+^	1.50*^+^	−2.17*^+^	−1.42*^+^
5	−17.00^∧^	−17.00^∧^	0.33^∧^	1.17*^+^	−0.75*	0.08
6	−30.00^∧^	−42.00*	0.58^∧^	1.00*	−1.00*^+^	−0.42^∧^

P = Participant. *Clinically significant change using the 2SD model, using the following values: MPA = 37.16; Flexibility = 0.96; Inflexibility = 0.70. ^+^Clinically significant according to recommended MDC for psychological flexibility (1.14) and inflexibility (0.90). ^∧^Change in the direction of functionality; however, not clinically significant.

As can be seen in Table 2, two individuals demonstrated clinically significant change in MPA at follow-up. One individual showed clinically significant change in psychological flexibility from pre- to post-treatment, and four participants’ change in psychological flexibility from pre-treatment to follow-up was clinically significant. For change in psychological inflexibility, three participants demonstrated clinically significant change from pre- to post-treatment, and two participants’ change was clinically significant from pre-treatment to follow-up. The majority of clinically significant changes in psychological flexibility and inflexibility as determined by the 2SD model were also supported by suggested MDC statistics.

### Follow-up

At 3-month follow up, all participants indicated they were continuing deliberate practice of skills learned at least once per week, with two participants practicing almost every day. Responses to open-ended questions reflected the ongoing use of mindfulness (*“I’ve been doing mindfulness nearly every morning”*), acceptance (*“[practicing] accepting thoughts and feelings”*), committed action (*“… asking myself about my willingness to do things”*), and self-observation (*“… the skill of identifying my own stress responses”*).

Five participants agreed or strongly agreed with the statement that skills learned in the program had helped with performances (one participant agreed somewhat). Responses to open-ended questions indicated that participants were integrating skills learned into performances and performance routines; for example, participants reported *“… it’s helped me to put my priorities in perspective,” “… this pattern of thinking has become more embedded in my thought processes before and during performances in particular,”* and *“I don’t have to think or focus on [ACT skills] as much because they become easier and more automatic [with time and practice].”* Comments also highlighted participants’ beliefs about the need for MPA interventions in this population. One participant wrote *“Please make this a subject at uni!!! SO MANY students could benefit from this…”* and another stated *“Thank you for providing this group to students and really caring about our mental wellbeing as performers!”*

## Discussion

This study sought to investigate whether group-based ACT may be a feasible and effective intervention for MPA in Australian student vocalists. Results showed that, at post-treatment and 3-month follow-up, participants demonstrated increased psychological flexibility, decreased psychological inflexibility, and decreased symptoms of MPA, compared to pre-treatment. Thus, hypotheses were supported, with the strongest effects observed for MPA. Transfer of treatment effects were also observed by reduced severity ratings for depression, generalized anxiety, and stress. While not statistically significant, change on these scales was clinically relevant with severity levels changing from moderate to normal levels (Lovibond and Lovibond, 1995). Wellbeing improved significantly from pre- to post-treatment, however, this was not maintained at 3-month follow-up. It is possible that being in a supportive group environment with others who understood their experiences improved participants’ wellbeing, such that the improvement was then not sustained when group sessions ceased. This may be an area for future investigation, as consideration may need to be given to ways to improve generalization of treatment gains at program cessation.

### Psychological Flexibility and Inflexibility

Psychological flexibility increased from pre- to post-treatment and continued on an upward trend from post-treatment to 3-month follow-up. This indicates a possible dose-response effect, in which case it may be beneficial to increase the number of sessions, for example, running a 1-h session per week in a 12-week program, as per the Eifert and Forsyth ACT for Anxiety treatment protocol (2005). It is also possible that more time invested in practice and utilization of skills leads to continued behavioral improvement, in which case it may be beneficial to offer booster sessions for a period of time post-treatment. Eifert and Forsyth (2005) highlight that while their program is designed as a 12-session weekly program, the number of sessions and how they are spaced should be kept flexible. It is also suggested there can be benefits in moving from weekly to monthly to quarterly booster sessions (Eifert and Forsyth, 2005). It is possible that spacing sessions out in such a way might also help to sustain improvements in wellbeing.

Psychological inflexibility decreased over the course of the intervention, being lowest at post-treatment. There was a slight trend towards an increase in psychological inflexibility at 3-month follow-up; however, scores remained lower than at pre-treatment. It is possible that participants did not generalize behaviors associated with psychological inflexibility to the extent that they did with behaviors associated with psychological flexibility. Examining the pattern of responses on the subscales of these measures provides insight into the construct validity of each these measures in relation to MPA. For example, the highest score reported on psychological inflexibility was *avoidance*, which is consistent with the proposition that individuals with SAD (of which MPA may be diagnosed as a subtype in DSM-5), avoid internal psychological and emotional experiences (thoughts and feelings) that occur in the context of social (performance) situations (Eifert and Forsyth, 2005). Yet, in contrast to the theorized interference of avoidance behaviors with values-guided action in SAD (Kocovski et al., 2013), the students in this study reported the highest psychological flexibility subscale scores for *committed action*. This is understandable given the high motivation and engagement of the students to participate in the program during the summer holiday break.

Acceptance and commitment therapy interventions directly target psychological flexibility and inflexibility, thus, the demonstrated changes in these scales were expected. This offers support for the fidelity of the intervention being consistent with ACT methodology, despite modifications to the program design to make it MPA-specific. Differences in the magnitude of change across psychological flexibility and inflexibility also offer support for the notion that they are two related yet distinct constructs (Rolffs et al., 2018), highlighting the importance of utilizing measures such as the MPFI to capture all 12 facets across both constructs.

### Music Performance Anxiety

Although symptom reduction is not a therapeutic focus in the ACT method, the significant decrease in MPA, which was maintained at 3-month follow-up, is notable. Despite reductions in MPA scores, however, no participants’ score fell below the generally accepted clinical cut-off of 105 (Kenny, 2016a). This cut-off was established on a sample of professional orchestral musicians who reported a mean K-MPAI score of 83.73 (*SD* = 40.72; Kenny et al., 2014a). Yet, the extremely high pre-treatment MPA scores reported in the current study are comparable to those across other studies utilizing diverse samples of musicians. Participants in the current study reported a mean pre-treatment MPA score of 140.33 (*SD* = 18.58), which is comparable with recent research utilizing diverse samples of musicians. For example, student vocalists (*M* = 143.71; Juncos et al., 2017), a sample (*N* = 100) of adult professional, amateur, and student classical musicians (*M* = 122.53, *SD* = 27.34; Kirsner, 2018), and a sample (*N* = 68) of musicians and singers aged 16–81 years with skill levels from beginner to semi-professional (*M* = 138.4, *SD* = 31.0; Kenny and Halls, 2018). This highlights a clear need to establish normative K-MPAI data and appropriate cut points for a more diverse sample of musicians, in particular student musicians.

Considering the high levels of MPA reported by students in this and other studies (e.g., Tamborrino, 2001; Kaspersen and Gotestam, 2002; Williamon and Thompson, 2006), there is a clear need for effective intervention in this population. Many young musicians report developing maladaptive coping, such as substance use, which can impact their wellbeing and career (Kenny, 2011; Braden et al., 2015), and it is uncommon for professional musicians to seek help from mental health professionals (Kenny et al., 2014a). Thus, this study adds to the body of literature recommending the importance of early intervention to enable young musicians to understand and manage their MPA early in their careers.

### Acceptance and Commitment Therapy for Music Performance Anxiety

There is increasing evidence for the effectiveness of ACT with a range of disorders (Australian Psychological Society, 2018), including SAD (Craske et al., 2014; Kocovski et al., 2013), and initial evidence for ACT as an effective intervention for MPA (Juncos and de Paiva e Pona, 2018). This study offers initial evidence for the feasibility and effectiveness of a group-based ACT intervention for MPA in students. Participants demonstrated improvements in MPA symptoms, psychological flexibility, and psychological inflexibility. Informal program evaluation suggested that participants continued to practice skills post-treatment and integrate them into performances and practice routines, indicating the intervention was acceptable to them.

### Limitations

As a pilot study, limitations included a small sample size (*N* = 6), restricted sample diversity, and lack of control group which collectively inhibit conclusions about causality and generalizability. This also limited ability to investigate processes of change (in particular the different effects psychological flexibility, inflexibility, and associated subscales). Thus, the research should be considered exploratory. Nevertheless, after applying Bonferroni adjustments the study still showed significant results across time and maintenance of some gains, particularly for reduced MPA. Other strengths of the research design included several confounding variables being controlled for (e.g., screening questions and interview controlled for current MPA treatment, mood disorders, and psychotic symptoms), manualized treatment consistent with recent ACT for MPA research, and oversight of treatment fidelity.

### Future Directions

Given the encouraging results with a small sample, the foundation is set for future research to extend into larger samples with greater diversity (e.g., inclusion of instrumental musicians at different stages of their training and career). Such studies could provide further evidence for the effectiveness of the intervention, enable investigation of change processes session-by-session, as well as deeper analysis of the sub-components of psychological flexibility and inflexibility. For instance, it is possible that participants’ avoidance was of internal experiences (e.g., thoughts, feelings, and somatic sensations) as opposed to overt behavior. Thus, participants may have been experiencing high levels of distress and avoidance while still committing to the valued action of music performance. Future research might also investigate the feasibility of incorporating ACT for MPA interventions into existing curricula at tertiary music education institutions. Given adherence and positive feedback provided in the informal program evaluation, it is likely future projects and programs would be well received. Lastly, given the wide use of the K-MPAI, there is a clear need to establish normative data and clinical cut-off points in more diverse samples, in particular student musicians.

## Conclusion

Given the extreme MPA scores reported by students, there is a clear need for appropriate and effective MPA interventions within this population. The current study offers initial evidence for the feasibility and effectiveness of a group-based ACT intervention for MPA with students. It is possible that such an intervention could be incorporated into existing tertiary music courses; the feasibility of this is a main area for future investigation.

## Data Availability Statement

The datasets generated for this study will not be made publicly available. The small sample size may compromise anonymity with certain tabulations of demographic variables.

## Ethics Statement

The studies involving human participants were reviewed and approved by The University of Melbourne Human Research Ethics Committee. The participants provided their written informed consent to participate in this study.

## Author Contributions

All authors designed the study. MO and LC obtained IRB ethics approval and advertised the study. MO obtained faculty support to host the program and financial reimbursement for participants. LC recruited and evaluated participants for the program. LC and MO designed and developed program materials and delivered the intervention with JB providing clinical supervision to ensure ACT integrity. LC conducted statistical analyses of the data with MO and JB providing research supervision. LC submitted this manuscript as part of her Masters in Psychology (Clinical) research study, supervised by MO and JB. All authors contributed to the final manuscript.

## Conflict of Interest

MO was employed as a Lecturer in Music (Performance Science) at the Melbourne Conservatorium of Music at the time of the study, although she was not directly involved in teaching any of the participants in the program. The remaining authors declare that the research was conducted in the absence of any commercial or financial relationships that could be construed as a potential conflict of interest.

## Footnotes

1 While the aim was to begin with 12 participants in the group program, only a small number of suitable participants were recruited. Following recommendations of between 5 and 7 for group-based interventions (Borek and Abraham, 2018) and 5 to 10 for group therapy (Yalom and Leszcz, 2005), six participants was deemed an acceptable number to run the study.
